# Evidence of Anti-Proliferative Activities in Blue Mussel (*Mytilus edulis*) By-Products

**DOI:** 10.3390/md11040975

**Published:** 2013-03-27

**Authors:** Lucie Beaulieu, Jacinthe Thibodeau, Claudie Bonnet, Piotr Bryl, Marie-Elise Carbonneau

**Affiliations:** 1 Department of Biology, Chemistry and Geography, University of Quebec at Rimouski (UQAR), 300 Allée des Ursulines, Rimouski, QC G5L 3A1, Canada; E-Mails: jacinthe.thibodeau@merinov.ca (J.T.); claudie_bonnet@uqar.qc.ca (C.B.); 2 Institute of Nutraceuticals and Functional Foods (INAF), Laval University, Quebec City, QC G1V 0A6, Canada; 3 Merinov, Quebec Fisheries and Aquaculture Innovation Centre, 96 montee de Sandy Beach, office 1.07, Gaspe, QC G4X 2V6, Canada; E-Mails: piotr.bryl@merinov.ca (P.B.); marie-elise.carbonneau@merinov.ca (M.-E.C.)

**Keywords:** blue mussels, *Mytilus edulis*, anticancer, enzymatic hydrolysis, peptides

## Abstract

Shellfish waste components contain significant levels of high quality protein and are therefore a potential source for biofunctional high-value peptides. The feasibility of applying a pilot scale enzymatic hydrolysis process to whole *Mytilus edulis* and, by fractionation, recover hydrolysates presenting a biological activity of interest, was evaluated. Fractions were tested on four immortalized cancerous cell lines: A549, BT549, HCT15 and PC3. The 50 kDa fraction, enriched in peptides, presented anti-proliferative activity with all cell lines and results suggest a bioactive molecule synergy within the fraction. At a protein concentration of 44 µg/mL, the 50 kDa fraction induced a mortality of 90% for PC3, 89% for A549, 85% for HCT15 and of 81% for BT549 cell lines. At the low protein concentration of only 11 µg/mL the 50 kDa fraction still entails a cell mortality of 76% for A549 and 87% for PC3 cell lines. The 50 kDa fraction contains 56% of proteins, 3% of lipids and 6% of minerals on a dry weight basis and the lowest levels detected of taurine and methionine and highest levels of threonine, proline and glycine amino acids. The enzymatic hydrolysis process suggests that *Mytilus edulis* by-products should be viewed as high-valued products with strong potential as anti-proliferative agent and promising active ingredients in functional foods.

## 1. Introduction

Marine organisms are constantly exposed to a hard, competitive and aggressive environment. They have therefore, developed various protective and defense mechanisms such as, the production of bioactive molecules [1]. Over the past decades, several researches exploring the potential of the marine biomass were pursued. The discovery of unique toxins with biological activities of interest led the way for the development of pharmaceutical products and further investigations for new biomolecules [2]. 

As a protection against water pathogens, marine organisms rely on their innate immune system [3,4,5,6]. Sessile organisms, such as Mollusca, are particularly exposed to predation and pathogens, and are thus more likely to produce bioactive secondary metabolites to protect themselves [5,7,8].

In Canada, the production of blue mussels, *Mytilus edulis*, accounts for almost 70% of the shellfish industry [9]. Mussel farms are concentrated in Prince Edward Island, but are also flourishing in Newfoundland and Labrador, New Brunswick, Nova Scotia, Quebec and British Columbia [9]. In 2009, 24,000 tons of mussels were harvested in Canada [9]. This successful aquaculture industry faces great environmental challenges as the exploitation generates important volumes of by-products essentially consisting of shells, damaged mussels and non-commercialized size mussels. These by-products are generally either discarded or used as animal feed or fertilizers [10]. Shellfish waste residues are constituted of high quality proteins (10% to 23% w/w) and therefore are a source for potential peptide mining [6]. Valorization of blue mussel’s by-products and conversion into high value biofunctional ingredients could provide a solution for environmental problems associated with aquaculture waste disposal and generate new revenues for the industry [6]. 

Biofunctional peptides are protein fragments usually ranging in size from 2 to 20 amino acids residues [6] and are associated with numerous potential physiological functions including immunomodulatory, antimicrobial, antithrombotic, opioid agonists or antagonists and antihypertensive activity [11,12,13]. Furthermore, anticancer biopeptides were isolated from marine organisms such as sponges, ascidians, mollusks and soft corals [14]. Some bioactive peptides have demonstrated multifunctional activities based on their amino acid composition, their sequence, their structure and other factors including hydrophobicity, charge or microelement binding properties [1,15,16]. Several biofunctional peptides have been reported from fish sources, but crustacean and mollusk sources have yet to be extensively studied [6]. Protein hydrolysates and peptides from mollusks and crustaceans have demonstrated antioxidant activity *in vitro* [6]. Currently, natural compounds extracted from marine mollusks are the source of at least four structurally distinct anticancer agents in clinical trial belonging to polyketides, terpenes, steroids and peptides classes [2,8,14]. However, to our knowledge, no studies were realized on the anti-proliferative potential of blue mussels hydrolysates, enriched in peptides.

Marine biofunctional peptides may be created by one of three methods such as solvent extraction, enzymatic hydrolysis, and microbial fermentation of food proteins [1]. In food and pharmaceutical industries the enzymatic hydrolysis method is desired, it avoids the use of chemical and physical treatments and therefore reduces the risk of destroying valuable molecules but most of all it prevents the detection of residual organic solvents or toxic chemicals in the final products [1,17]. Enzymatic hydrolysis produces short chain peptides and free amino acids. Peptides may, within their original protein sequence, be inactive and their bioactivity be triggered by the enzymatic hydrolysis [1,7]. For example, it is reported that peptides have substantially higher antioxidant activity than intact proteins [13] and may demonstrate enhanced solubility, heat stability, water binding ability and increased nutritional quality [18]. 

In the present study, proteolytic hydrolysis and subsequent fractionation of whole blue mussels, *Mytilus edulis*, was performed according to a method developed at the fractionation center of Merinov, Gaspé [19]. The different fractions obtained were tested for anti-proliferative activity against four immortalized cell lines: A549 type II pulmonary epithelial cells, HCT15 colon carcinoma cells, BT549 breast carcinoma cells and PC3 prostate cancer cells. Cell growth inhibition was determined by a luminescence measurement method. The objective was to target the fractions with the highest inhibition activity on the different cancer cell lines and to do a preliminary characterization of the fractions of interest.

## 2. Results and Discussion

### 2.1. Processing of Blue Mussels

The aim of this research was to process whole blue mussels using enzymes and, by fractionation, recover fractions with anti-proliferative properties. A pilot scale batch process was performed with approximately 100 kg of raw material. The dry matter distribution (mass balance in %) was monitored throughout the process and is displayed in Table 1. Enzymatic hydrolysis was performed with Protamex, a commercial *Bacillus* protease complex with broad specificity to hydrophobic amino acids [20,21], known to be food-grade [22] and produce non-bitter hydrolysates [17]. Considering the raw material as 100%, 71.1% of the initial dry matter was contained in the residual solid mass from the hydrolysis, while 12.1% was recovered in the hydrolysate. Mussels’ shells are essentially formed of calcium carbonate [23,24,25,26]. The high content in calcium carbonate in the raw material could be a key parameter affecting the enzymatic hydrolysis efficiency [27] and could explain the high percentage of residual solid mass. Furthermore, in Autumn *Mytilus edulis* wet weight is evaluated to be 85% to 90% shell and 10% to 15% meat. Major loss of material occurred in this decantation phase, since only 81.3% of the original dry weight of the raw material was recovered. 

Following the centrifugation step, two fractions were obtained: a centrifugation pellet containing 5.2% of the total dry matter and a liquid phase containing 7.0% of the total dry matter. Experimental conditions could be modified in order to enhance the liquid separation. The liquid phase was fractionated in three retentates: 50 kDa, 1 kDa and 200 Da (nano-filtration) respectively corresponding to 1.4%, 0.8% and 2.8% of the total dry matter. As can be seen in Table 1, not all of the mussel dry matter was recovered after the hydrolysis and fractionation steps. The cumulative recovery was 81.3%. Some of the process steps could have been a factor in these major losses. The process was performed in a pilot plant, using equipment that was not optimized to maximize hydrolysate recovery. Dead volumes in the equipment, such as the separators and pipes, might have trapped some of the hydrolysate. Furthermore, small aliquots at the beginning of each separation step were taken for analyses. In the present work, the solid fractions obtained following the decantation and centrifugation steps corresponded to 76.3% of the whole blue mussels’ dry weight raw material. This material, composed principally of shells residues, can find utilization for animal feed additive or constituents in fertilizer [26,28,29,30]. Essentially made of calcium carbonate, mussels shells residues could also be used as liming agent in acid soils, for the recovery of mine tailings [26] or even as a potential limestone or sand substitute for agricultural, construction and engineering purposes [29]. The other 7.0% of the mass is regarded as material of human nutritional interest, rich in both proteins and minerals (Table 2). 

**Table 1:** Mass balance of fractions obtained by processing approximately 100 kg of blue mussels (results of a representative process performed in triplicate in 2008).

Fractions	Wet weight (kg)	Dry weight (kg)	Mass balance ^A^ (%)
Crude extract (1:1)	150	82.85	100
Solid after sieving	73.50	58.87	71.06
Liquid after sieving	190	10.05	12.13
Solid after centrifugation	21.75	4.33	5.22
Liquid phase	170	5.80	7.00
50-kDa ultra-filtration retentate	25.20	1.15	1.39
1-kDa ultra-filtration retentate	26.50	0.64	0.77
Nano-filtration retentate	31.80	2.33	2.81
*Cumulative recovery* ^B^			81.25

^A^ Expressed as % on a dry matter basis. ^B^ Cumulative recovery was calculated based on the mass balance (%) of all resulting fractions (The values associated to both liquid after decantation, and liquid after centrifugation, were not taken in account in this calculation since these fractions were used as starting steps further in the process).

**Table 2:** Chemical composition of fractions obtained by processing of approximately 100 kg of blue mussels.

Fractions	Dry matter ^A^ (%)	Proteins ^A^ (%)	Lipids ^A^ (%)	Minerals ^A^ (%)
Crude extract (1:1)	55.23 ± 1.64	10.74 ± 0.37	0.97 ± 0.07	79.92 ± 1.52
Solid after sieving	80.10 ± 1.39	5.84 ± 0.04	0.27 ± 0.02	91.29 ± 1.15
Liquid after sieving	5.29 ± 0.04	56.22 ± 4.76	8.03 ± 0.60	28.75 ± 3.97
Solid after centrifugation	19.91 ± 0.08	45.73 ± 0.20	11.08 ± 0.44	24.13 ± 1.57
Liquid phase	3.41 ± 0.00	57.77 ± 2.07	4.55 ± 1.45	21.49 ± 0.00
50-kDa ultra-filtration retentate	4.56 ± 0.01	56.42 ± 0.09	3.18 ± 0.15	5.71 ± 0.32
1-kDa ultra-filtration retentate	2.42 ± 0.00	70.45 ± 0.29	4.55 ± 1.75	21.49 ± 0.00
Nano-filtration retentate	7.32 ± 0.00	67.55 ± 0.29	0.55 ± 0.39	15.78 ± 0.29

Results are mean values of two replicates ± SD. ^A^ Expressed as % (g per 100 g) on a dry weight basis.

### 2.2. Chemical Composition of Fractions

All fractions obtained from the entire fractionation process were characterized. The moisture, mineral, protein and lipid contents were determined. Table 2 presents the overall chemical composition on a dry weight basis. The crude extract, obtained after grinding, contained 44.8% of water, while the solid portion contained almost 80% of minerals. Mollusca shells are multilayered organo-mineral structures of which the mineral portion, calcium carbonate, accounts for 95% to 99% by weight [23,24,25,26]. The remaining 1%–5%, the organic matrix, is essentially made of proteins [24,26]. The crude extract, including whole blue mussels, contained only 10.7% of protein and 1.0% of lipids.

Depending on the subsequent steps included in the processing of blue mussels, the components were recovered in varying proportions. The solids left after decantation were mainly composed of minerals (91.3%), primarily associated with the mussels shells. The protein and lipid contents were 5.8% and 0.3%, respectively. Mussels are low lipid meats [31]. Depending on the season, between 0.5% to 2.5% wet weight of soft tissue is lipid [32]. Higher lipid content is generally associated with the spawning season [33]. In comparison, the mackerel is more than 39% rich in lipids and the snow crab presents a concentration of around 5% of lipids [19,34].

The dry matter in the liquid after decantation was rich in protein (56.2%) and minerals (28.8%) and contained a small amount of lipids (8.0%). The purpose of the centrifugation step in the bioprocess was to remove all solid particles in suspension. Two fractions were obtained, the solid and the liquid phase. Solids were principally composed of protein (45.7%) and minerals (24.1%) but also 11.1% of lipids.

The aqueous phase was slightly more concentrated in proteins (57.8%) and contained less minerals (21.5%) and lipids (4.6%). The aqueous phase underwent further fractionation using different filtration steps. Many of the minerals and some of the amino acids are hydrophilic compounds that might be enriched in the aqueous fraction [35]. The first steps involved using a micro-filtration membrane followed by an ultra-filtration membrane with a 50-kDa cut-off. 

The dry matter in the 50 kDa retentate thus obtained was composed of 56.4% protein, 3.2% lipid and 5.7% minerals. A significant amount of protein was retained in this step, consisting likely of proteins of higher molecular weight. Even if the lipid concentration detected in the 50 kDa fraction was low, some proteins could have form complexes with polar lipids and thus were retained [36]. Both the 1 kDa and the nano-filtration retentates contained solids consisting mainly of proteins (70.5% and 67.6%, respectively) as well as minerals (21.5% and 15.8%, respectively). The 1 kDa and nano-filtration retentates were the most enriched in protein suggesting high concentration of small peptides and free amino acids in the blue mussels’ fractionation aqueous phase. The concentration of lipids in the nano-filtration fraction was almost inexistent; all lipids were retrieved in the 1 kDa fraction (4.6%).

### 2.3. Amino Acid Analyses

The protein enriched fractions; the liquid phase, the 50 kDa, the 1 kDa and the nano-filtration retentates, were also analyzed for their amino acid contents (Table 3). Data in Table 3 are divided into commonly considered, essential and nonessential amino acids groups and expressed as % (g/100 g), on a dry weight basis. The amount of essential amino acid component differed depending on the fraction analyzed. All fractions had high content in lysine and leucine with highest values detected in the nano-filtration retentate: 2.8% ± 0.2% and 2.6% ± 0.0%, respectively. While lysine is reported to contribute to mussels’ adhesion via ionic bonding to negatively charged surfaces [37], leucine, as well as histidine, tyrosine, methionine and cysteine, is associated with a radical scavenging activity [6]. The highest threonine and serine contents were detected in the 50 kDa fraction (2.2% ± 0.1% and 1.9% ± 0.0%, respectively). Phosphate ester bonds are hydrolyzed under acidic conditions contributing to the observation of free serine and threonine amino acids [38]. Threonine, as well as serine, is known to be involved in the reproduction mechanisms of bivalve mollusk [39]. The liquid phase, the 50 kDa, the 1 kDa and the nano-filtration fractions were made up of approximately 30.5%, 31.0%, 28.1% and 34.3%, of essential amino acids respectively. 

**Table 3:** Total amino acids in the fractions obtained from processing of blue mussels.

Amino acid ^A^	Centrifugation aqueous phase	50-kDa ultra-filtration	1-kDa ultra-filtration	Nano-filtration
***Essential***				
Histidine	0.79 ± 0.04	1.09 ± 0.04	0.91 ± 0.07	1.07 ± 0.18
Isoleucine	1.30 ± 0.04	1.41 ± 0.01	1.37 ± 0.13	1.82 ± 0.03
Leucine	1.90 ± 0.16	1.99 ± 0.03	1.99 ± 0.12	2.63 ± 0.00
Lysine	2.20 ± 0.34	2.48 ± 0.11	2.68 ± 0.08	2.80 ± 0.24
Methionine	0.65 ± 0.15	0.37 ± 0.04	0.71 ± 0.04	0.79 ± 0.01
Phenylalanine	0.99 ± 0.09	1.18 ± 0.06	0.92 ± 0.07	1.59 ± 0.33
Threonine	1.45 ± 0.02	2.24 ± 0.07	1.78 ± 0.08	1.80 ± 0.32
Tryptophan	n.a.^C^	n.a. ^C^	n.a. ^C^	n.a. ^C^
Valine	1.46 ± 0.05	1.75 ± 0.04	1.61 ± 0.16	1.95 ± 0.03
**Total (a)**	**10.74**	**12.51**	**11.97**	**14.45**
***Nonessential***				
Alanine	2.00 ± 0.12	1.94 ± 0.01	2.32 ± 0.10	2.12 ± 0.11
Arginine	2.53 ± 0.05	2.27 ± 0.10	2.80 ± 0.24	3.86 ± 0.17
Aspartic acid	3.59 ± 0.41	5.08 ± 0.11	5.65 ± 0.08	4.04 ± 0.05
Cysteine	0.38 ± 0.02	0.67 ± 0.10	0.88 ± 0.13	0.25 ± 0.03
Glutamic acid	4.96 ± 0.32	6.81 ± 0.09	7.52 ± 0.18	5.37 ± 0.08
Glycine	3.63 ± 0.04	5.00 ± 0.17	4.21 ± 0.24	3.06 ± 0.34
Proline	1.60 ± 0.01	2.95 ± 0.11	2.36 ± 0.16	1.58 ± 0.07
Serine	1.51 ± 0.07	1.94 ± 0.01	1.90 ± 0.04	1.67 ± 0.16
Taurine ^B^	3.39 ± 0.05	0.58 ± 0.03	1.94 ± 0.07	4.27 ± 0.53
Tyrosine	0.91 ± 0.01	0.59 ± 0.03	1.10 ± 0.03	1.51 ± 0.32
**Total (b)**	**24.50**	**27.83**	**30.68**	**27.73**
**Grand total (a + b)**	**35.24**	**40.34**	**42.65**	**42.18**

^A^ Expressed as % (g/100 g) on a dry matter basis. ^B^ Taurine is a sulfur-containing amino acid derived from methionine and cysteine. ^C^ n.a., not analyzed.

In general for all fractions, the nonessential amino acid group was rich in aspartic acid, glutamic acid and glycine. It should be noted that asparagine and glutamine are amide derivatives of aspartic acid and glutamic acid, respectively. During acid hydrolysis, which cleaves amide bonds, asparagine is converted to aspartic acid and glutamine to glutamic acid. Thus, the amount determined for aspartic acid represents the total of aspartic acid and asparagine and similarly for glutamic acid and glutamine [40]. Proline, involved in the adhesion of mussels [41], was higher in the 50 kDa and the 1 kDa fractions. Proline, an hydrophobic amino acid, is also linked to the inhibition of lipid peroxidation [6]. High concentrations of glycine, also a constituent of mussels’ adhesive proteins, were also found in all fractions with the highest observation in the 50 kDa fraction with 5.0% of glycine. Glycine, taurine and alanine are the most representative amino acids in volume regulation of bivalves [42]. High concentration of taurine was found in the liquid phase and the nano-filtration fraction. Taurine is an amino acid, not utilized in protein synthesis, but rather is found free or in simple peptides. It is known to be highly concentrated in seafood especially which derived from invertebrates such as mollusks and crustaceans [6,43]. Taurine may be a pertinent candidate for use as a nutritional supplement to protect against oxidative stress, neurodegenerative diseases or atherosclerosis [43]. In mussels, taurine is the main contributor in osmotic adaptation [44] and is commonly associated with gill tissue [45]. Methionine and cysteine, precursors of taurine, were found in trace amounts [46]. Calcium carbonate could diminish the efficiency of enzymatic hydrolysis [27]; the removal of mussels’ shells could therefore, lead to a better recovery yield of amino acids.

### 2.4. Anti-Proliferative Activities

Anti-proliferative activity was shown by a decrease in cancerous cells A549, BT549, HCT15 and PC3 growth. Preliminary tests performed with the liquid phase, the 50 kDa, the 1 kDa and the nano-filtration retentates showed that only the 50 kDa fraction presented anti-proliferative activities with all cancerous cell lines tested. 

**Figure 1:**
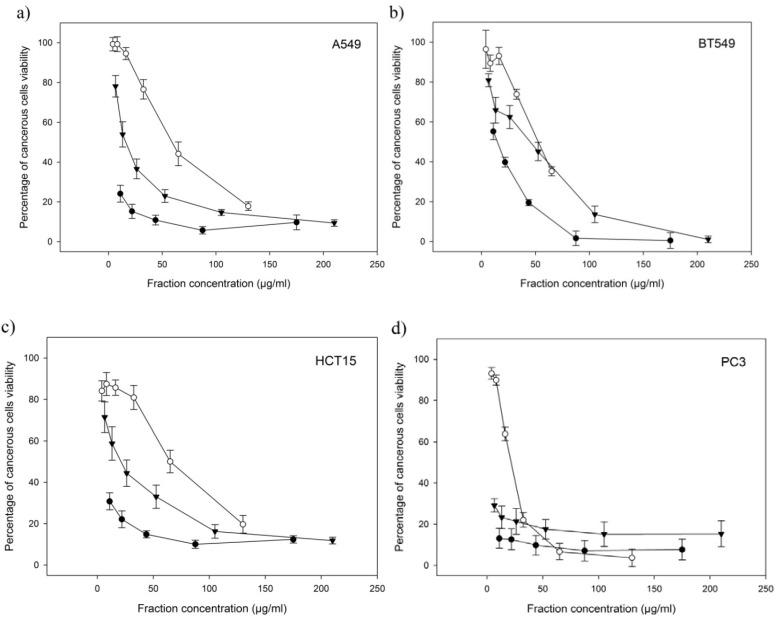
Percentage of cell viability (cell growth) obtained for each cancerous cell line after a 72 h exposure with the 50 kDa fraction and both A and B sub-fractions. Results are mean values of four replicates ± SE. (**a**) A549. (**b**) BT549. (**c**) HCT15. (**d**) PC3. As a positive control, Etoposide at a concentration of 60 μM, displayed a cells viability of 0% for A549 cells, of 0% for BT549 cells, lower than 10% for HCT15 cells and lower than 25% for PC3 cells (for *n* = 3). The black circles represent the results for the 50 kDa fraction; the empty circles, for the A sub-fraction and the black triangles, for the B sub-fraction.

In order to recover fractions with anti-proliferative activity, the 50 kDa retentate was purified using a cation exchange column. Two sub-fractions, A and B, were obtained. Figure 1a–d shows the different percentage of cell growth obtained for each cancerous cell line after an exposure of 72 h with the 50 kDa fraction and both A and B sub-fractions.

At concentrations below 50 µg/mL, the 50 kDa and B fractions entailed an A549 cancerous cell inhibition growth of more than 50%. At a concentration of 26 µg/mL, the B fraction caused 63.4% ± 4.9% of cell inhibition growth. At a concentration of 22 µg/mL, the 50 kDa fraction induced an A549 pulmonary epithelial cancer cells inhibition growth of 84.8% ± 3.4% and of 75.9% ± 4.2% at 11 µg/mL. Even at really low concentrations, the 50 kDa fraction clearly demonstrated a higher anticancer efficiency against A549 cell line in comparison to A or B fractions. 

At concentrations below 50 µg/mL, only the 50 kDa fraction caused an inhibition growth of more than 50% to BT549 breast carcinoma cell line. The 50 kDa fraction induced an inhibition growth of 80.5% ± 1.5% at a concentration of 44 µg/mL and of 60.2% ± 2.4% at 22 µg/mL. The 50 kDa fraction was less efficient against BT549 than against A549 cell line but still showed a higher anti-proliferative efficiency than A or B fractions.

At concentrations below 50 µg/mL, the 50 kDa and B fractions produced a HCT15 colon carcinoma cell inhibition growth of more than 50%. The B fraction induced a cell inhibition growth of 55.6% ± 6.4% at a concentration of 26 µg/mL. The 50 kDa fraction caused a cancerous cell inhibition growth of 83.1% ± 1.7% at a concentration of 44 µg/mL, of 77.9% ± 4.1% at 22 µg/mL and of 69.2% ± 4.1% at 11 µg/mL. Against HCT15 cell line the 50 kDa fraction exhibited a higher anti-proliferative efficiency than A or B fractions but was less efficient against HCT15 than against A549 cell line.

At concentrations below 50 µg/mL, all fractions entailed a PC3 prostate cancer cell mortality of more than 50%. The A fraction induced 77.9% ± 3.6% at a concentration of 33 µg/mL, the B fraction occasioned an inhibition growth of 78.6% ± 6.2% at 26 µg/mL 76.6% ± 5.5% at 13 µg/mL and of 70.8% ± 3.2% at 6.5 µg/mL. The 50 kDa fraction produced a cancerous cell inhibition growth of 90.2% ± 4.8% at 44 µg/mL, of 87.3% ± 5.1% at 22 µg/mL and of 86.8% ± 4.8% at 11 µg/mL. Overall, the 50 kDa fraction still entailed a higher anti-proliferative efficiency than A or B fractions. The 50 kDa fraction was more efficient against PC3 than against A549, BT549 and HCT15 cell lines.

Preliminary results show that the 50 kDa hydrolysate fraction, enriched in peptides, possessed anti-proliferative properties with all tested cell lines. More specifically, it demonstrated high anti-proliferative activity towards PC3 prostate cancer cell line and A549 pulmonary epithelial cancer cells. Moreover, the 50 kDa fraction is clearly more efficient against all four cancerous cell lines than the purified A and B fractions. Those results suggest a potential synergy of bioactive molecules such as peptides within the 50 kDa fraction not encountered in purified extracts; as a matter of fact the purification of bioactive molecules including peptides leads to fractions possessing weaker anti-proliferative activity. 

The National Cancer Institute (U.S.) indicates that an extract, to be commercially valuable, must present a biological efficiency at concentration below 100–150 µg/mL. Furthermore the Marine Biotechnology Research Center (personal communication, MBRC, Quebec, Canada) specifies that only biomolecules expressing a biofunctional activity at concentrations below 10 µg/mL are worth further pharmaceutical investigations and are of potential commercial interest. Following those guidelines, we concluded that the 50 kDa fraction, entailing more than 75% A549 and 80% PC3 cancerous cell mortality at a concentration of 11 µg/mL, possesses a strong potential as an effective anti-proliferative agent against both of those cancerous cell lines. 

## 3. Experimental Section

### 3.1. Processing of Blue Mussels

Whole blue mussels (*Mytilus edulis*) were harvested in autumn and supplied by Menu-Mer Ltée (Gaspe, Canada) grown in an area certified safe by Fisheries and Oceans Canada. Mussels provided consisted of industrial waste: damaged shells or non-commercial size blue mussels (below 60 mm). *Mytilus edulis* hydrolysate fractions were produced at the Quebec Fisheries and Aquaculture Innovation Centre (Merinov, Gaspe, QC, Canada) according to the procedure described previously [19,34]. Briefly, 100 kg of grinded entire blue mussels was added to 160 kg of demineralized water, the total volume was heated to 45 °C and 100 g Protamex^®^ (Novozymes, Bagsvaerd, Denmark) was added to start the hydrolysis. The mixture was constantly mixed during the hydrolysis. After 60 min hydrolysis at 45 °C, the tank temperature was increased to 90 °C, to inactivate the proteases. The liquid portion was decanted using a clarifying decanter (gravity force around 3500 *g*) and then centrifuged (gravity force of 11,000 *g*) in order to separate suspended insoluble matter and lipids from the hydrolysate [19]. The hydrolysate was ultrafiltered (spiral membranes with cut-offs of 50 and 1 kDa) to separate the proteins and peptides by molecular mass. Permeate from the 1 kDa membrane was nano-filtered and run through a pilot scale reversed osmosis system (Model R, GEA filtration, Hudson, WI, USA). Protein hydrolysates were therefore separated into three major classes: 50 kDa retentate (>50 kDa), 1 kDa retentate (50–1 kDa) and nano-filtration retentate (1 kDa–200 Da). Final fractions were kept frozen until analyses (−20 °C). This entire process, allowing the fractionation of whole blue mussels, was first performed in 2006 and was repeated and optimized in 2008. A sieving step replaced the decantation step. Results presented in this article are results from the processing of three batches performed in 2008 at pilot scale (approximately 100 kg) at the Quebec Fisheries and Aquaculture Innovation Centre (Merinov, Gaspe, QC, Canada).

### 3.2. Fraction Chemical Composition Determination

Fractions obtained from whole blue mussels fractionation process were characterized to determine their chemical composition. Moisture and minerals (ash) were measured using the official methods of analysis of the Association of Official Analytical Chemists [47]. Lipids were analyzed using a modified Bligh and Dyer method [48]. Proteins were determined by the Kjeldahl method (nitrogen_6.25) adapted from the official method of AOAC 988.05 [47].

### 3.3. Amino Acid Analyses

Amino acid determination of protein hydrolysates was performed using the AccQ-Tag amino acid analysis procedure (Waters, Mississauga, ON, Canada) for determination of amino acids resisting to acidic hydrolysis (including taurine). The AccQ-Tag method is a pre-column derivatization technique for peptide and protein hydrolysate. 

Amino acids were separated by reversed-phase high performance liquid chromatography (RP-HPLC) and quantified by fluorescence detection. The HPLC system used was equipped with a Waters Alliance e2695 Separations Module (Waters, Mississauga, ON, Canada) and a Waters 2475 Multi λ Fluorescence Detector. Amino acid analysis of blue mussel fractions (previously lyophilized) was performed using a Waters AccQ-Tag Amino Acid Analysis Column (silica base bonded with C_18_, Waters, Mississauga, ON, Canada). The column was calibrated using Amino acid standard H (Pierce, Rockford, IL, USA) and taurine (Sigma, Oakville, ON, Canada) as reference standards. Based on the AccQ-Tag method which incorporates a 45 min non-linear gradient including several steps, optimization was performed to allow taurine separation and quantification. The method started with 100% of Eluent A (aqueous buffer, Waters AccQ-Tag Eluent A, Mississauga, ON, Canada) and decreased to 95% of Eluent A in combination with 5% of Eluent B (HPLC-grade acetonitrile) in 19 min. This condition was maintained up to 24 min. Then, the Eluent A decreased to 85% while the Eluent B increased to 15% up to 27 min. From 27 to 29.5 min the Eluent A decreased to 83% and Eluent B increased to 17%. Finally, from 33 to 45 min, the Eluent proportions were 60% of Eluent B in combination with 40% Eluent C (NANOpure water, Barnstead, Dubuque, IA, USA). A volume of 5 µL of samples and standards were loaded on the column and eluted at a flow rate of 1 mL/min at a column temperature of 37 °C. Amino acids analyzed on the Waters AccQ-Tag column (Waters, Mississauga, ON, Canada) were detected by fluorescence. Data were collected and integrated using the Empower 2 software. 

### 3.4. Anti-Proliferative Activities

Fractions were tested for anti-proliferative activity at the Marine Biotechnology Research Center (MBRC, Quebec, Canada) according to the following protocol.

#### 3.4.1. Cell Culture

Cell lines, A549 (type II pulmonary epithelial cell), HCT15 (colon carcinoma cell), BT549 (breast carcinoma cell) and PC3 (prostate cancer cell) were cultured in F12K and RPMI1640 medium (HyClone, UT, USA) supplemented with 10% (v/v) fetal bovine serum (FBS) (HyClone, UT, USA), 100 U/mL penicillin and 100 mg/mL streptomycin (GIBCO Inc, Grand Island, NY, USA) at 37 °C in a humidified atmosphere with 5% CO_2_. 

#### 3.4.2. Sulforhodamine B (SRB) Assay

Cells were cultured in a 96-well plate at a density of 1 × 10^5^ cells per mL. The cells were then treated with a 1:2 serial dilution of the fractions obtained from processing of mussels: liquid phase, 50 kDa, 1 kDa, nano-filtration retentates, A and B sub-fractions obtained from the former 50 kDa fraction. Their starting concentrations were 119, 350, 66, 263, 130 and 210 µg/mL, respectively. After 3 days (72 h), the cells were treated with the anionic dye sulforhodamine B (Sigma-Aldrich) [49,50]. The amount of luminescence was directly proportional to the number of living cells in cultures [51,52,53]. All conditions were performed in duplicate wells and in two independent assays.

In this assay, positive control was the apoptotic agent Etoposide (300 μM; Sigma-Aldrich) which was tested at different concentrations (1/5, 1/25, 1/125, 1/625 and 1/3125). The negative controls were the sterile water which is considered as the vehicle condition (solvent used to dissolve the tested compounds), and the culture medium which is considered as the basal condition. 

### 3.5. Purification of Tested Fractions

Several ion exchange chromatography (IEX) and hydrophobic interaction chromatography (HIC) resins were tested using HiTrap™ IEX Selection Kit and HiTrap™ HIC Selection Kit (GE Healthcare, Baie d’Urfe, QC, Canada). A scale up was performed to reproduce the purification step with the fraction that demonstrated anti-proliferative activity. The active fraction was captured using a SP-Sepharose™ Fast Flow cation exchange column (GE Healthcare, Baie d’Urfe, QC, Canada) containing 20 mL of resin. The column (GE Healthcare, Baie d’Urfe, QC, Canada) was connected to a FPLC system (AKTÄ Explorer 100, GE Healthcare, Baie d’Urfe, QC, Canada). The system was equipped with UV (215, 254, 280 nm), conductivity and pH monitors, which were connected to an autosampler and a fraction collector. The chromatograms were obtained and analyzed using the UNICORN 5.0 software. The column was equilibrated with 5 CV (CV = column volume) of 50 mM sodium phosphate buffer at pH 7 (Buffer A). The peptide solution was applied to the column at a rate of 156 cm/h; then, the column was washed successively with Buffer A alone and Buffer A containing 1 M NaCl. Bioactive fractions were eluted from the column with Buffer A. Purified fractions were also tested for anti-proliferative activities as described previously.

## 4. Conclusions

This work is a part of a global project which includes the study of anti-proliferative activity in different biomasses exploited in Quebec. Only fractions from mussels showed significant anti-proliferative activities, while other biomasses such as mackerel and herring were also subjected to hydrolysis by Protamex [34,54]. The aim of our work was to target the fractions, enriched in proteins/peptides, with the highest inhibition activity on the different cancer cell lines and to do a preliminary characterization of the fractions of interest. Enzymatic hydrolysis using proteases was performed in order to generate bioactive peptides of interest.

The enzymatic hydrolysis process described in the present study, despite non-optimized conditions, gave interesting results as fractions demonstrated *in vitro* anti-proliferative activity. Optimization of the process could lead to a greater yield of the entire process and isolation of fractions with enhanced anti-proliferative activity. For example, the use of different enzymes may be a solution to improve the cumulative recovery. Furthermore, calcium carbonate was reported to affect the enzymatic hydrolysis [27], removal of mussels shells may therefore, increase the efficiency and enhance the recovery of bioactive peptides. The enzymatic hydrolysis of mussels harvested in the spring rather than autumn could also positively affect the anti-proliferative potential of recovered biofunctional peptides. Higher levels of triglycerides and phospholipids in *Mytilus edulis* are associated with the Spring spawning season [33,55] and polyunsaturated fatty acids, such as omega-3, have demonstrated inhibitory effect against several human cell lines derived from colonic, pancreatic, prostate and breast cancer [56].

Of all fractions obtained and purified, the specific 50 kDa hydrolysate exhibited the highest anti-proliferative activity on all four immortalized cancerous cell lines. The inhibition activity was particularly strong with PC3 prostate cancer cells and A549 type II pulmonary epithelial cancer cells, with respectively around 87% and 76% of inhibition growth for only 11 µg/mL of hydrolysate. Results suggest a possible synergy of bioactive molecules such as peptides within the 50 kDa hydrolysate, leading to higher anti-proliferative activity than in subsequent purified fractions. Further analyses are required to identify the specific bioactive molecules including peptides responsible of the strong anti-proliferative activity and confirm a potential bioactive peptides synergy. 

Evidence suggests that peptides derived from the enzymatic hydrolysis of *Mytilus edulis* by-products have potential health benefits and promising applications as active ingredients for functional foods or nutraceutical and pharmaceutical products. The high nutritive potential of mussels as a source of protein, vitamin C, iron, zinc and omega-3 is well documented [31]. However, further research on large-scale production, gastrointestinal stability, bioavailability and long term stability need to be performed [6]. Moreover, efficiency and bioactivity of marine derived peptides have essentially been tested *in vitro* or in animal models and reports of clinical researches on human are very limited [6]. 
